# Stress May Drive Plant Patterns

**DOI:** 10.1371/journal.pbio.1000517

**Published:** 2010-10-19

**Authors:** Robin Meadows

**Affiliations:** Freelance Science Writer, Fairfield, California, United States of America

**Figure pbio-1000517-g001:**
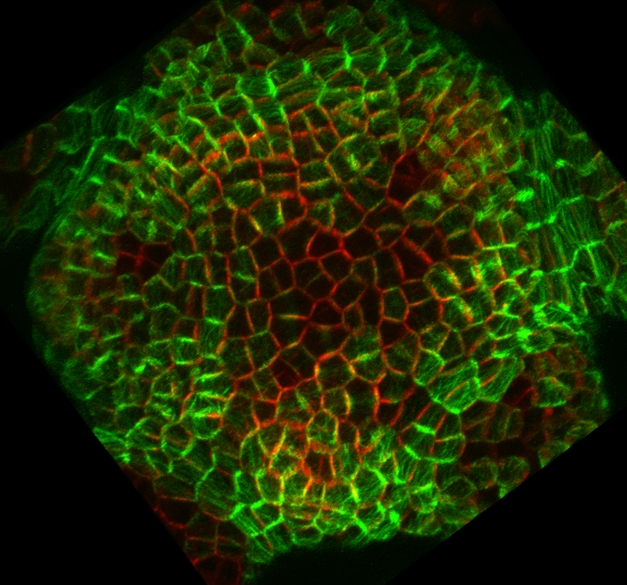
In the *Arabidopsis* shoot meristem, the orientation of cellular growth and the direction of auxin transport are coupled through a common orientation of interphase microtubules (green) and PIN1 auxin efflux carrier localization (red).

[Fig pbio-1000517-g001]Plants grow in a wondrous assortment of patterns, from simple to complex, with near mathematical precision. Honeysuckles sprout leaf pairs at regular intervals along their stems, for instance, whereas some succulents form double spirals turning in opposite directions. The big question is how do they do this? New research reported in this issue of *PLoS Biology* suggests that mechanical stress is at the root of plant patterns, challenging the longstanding theory that the plant hormone auxin acts alone to direct this patterning.

Regardless of the growth pattern, new leaves and flowers arise from blobs of pluripotent cells at shoot tips (the shoot apical meristem, or SAM). Auxin triggers the transformation of these shoot tip cells into new organs and is also linked to plant growth patterns—when a given SAM cell has high auxin levels, the neighboring cells form clusters of an auxin export protein (PINFORMED1 or PIN1) in the parts of their plasma membranes that border the auxin-rich cell. This sets up an auxin circulatory system in which the auxin-rich cells get even more of this hormone, further boosting their growth.

But although this chemical signaling could drive plant patterning in theory, it is difficult to see how it would work in actuality. Because auxin lacks directional effects in cells, it is unlikely to cause PIN1 clustering, leaving the origin of plant designs a mystery. Recently, however, Marcus Heisler and colleagues showed that mechanical stress is linked to the direction of organ growth, and here they reveal that stress is also linked to plant patterning.

Whereas auxin initiates leaf and flower development, the direction of this new growth depends on the orientation of microtubules in cell cytoskeletons. Plant plasma membranes are encased in sturdy cell walls that contain load-bearing components, such as cellulose, and chemical treatments that modify the mechanical properties of these walls can disrupt plant patterning. The researchers had previously found that when cell walls of the small flowering plant *Arabidopsis thaliana* are perturbed mechanically, the orientation of shoot tip microtubules reflects the resulting mechanical stress patterns.

To see if stress also directs PIN1 clustering in shoot tips, the researchers treated *Arabidopsis* with isoxaben, which weakens cell walls by inhibiting cellulose synthesis. Treated shoot tip cells keep growing despite the absence of additional cellulose, which presumably increases the stress on their cell walls. The researchers then visualized the distributions of microtubules and PIN1. Before isoxaben treatment, microtubules were randomly oriented in many shoot tip cells, whereas after treatment, these cytoskeletal components formed thick bundles that were aligned as expected relative to increased stress. Likewise, PIN1 clustering in isoxaben-treated cells also matched the predicted stress patterns. For example, PIN1 was predominantly in cell corners, and corners are associated with high stress in structures.

As an additional test of whether mechanical forces could generate these observed PIN1 patterns, the researchers developed a mathematical model accounting for stress, auxin transport and PIN1 dynamics. The model confirmed that cell wall stress could generate PIN1 distribution patterns, which could in turn generate plant growth patterns that, like those found in nature, are periodic.

The researchers propose that patterning depends on the following chain of events: as a given shoot tip cell expands, stress levels in the adjacent cell walls of neighboring cells increase. This causes PIN1 clustering in the adjacent plasma membranes of neighboring cells, which then export auxin to the expanding cell, which then grows even larger. Periodic PIN1 distribution patterns would then give rise to periodic shoot tip cell growth, thus creating the overall pattern of leaf or flower growth.

Besides accounting for plant patterning just as well as the chemical signaling model, this new mechanical signaling model has the added benefit of explaining additional observations. For example, root formation can be induced mechanically in *Arabidopsis*. And flower primordium initiation can be induced by pectin methyl-esterase, which likely changes the viscoelastic properties of cell walls by altering the degree of pectin cross-linking.

Although it will be important to test this mechanical model further experimentally, this work makes a compelling case that a common mechanism—stress—drives both the microtubule orientations and the PIN1 polarities critical to the beautiful patterns of leaves and flowers in the world around us. It will be interesting to investigate other possible roles for mechanical signaling in development as well as the coordination of growth localization and growth direction in both normal development and wound repair.

**Heisler MG, Hamant O, Krupinski P, Uyttewaal M, Ohno C, et al. (2010) Alignment between PIN1 Polarity and Microtubule Orientation in the Shoot Apical Meristem Reveals a Tight Coupling between Morphogenesis and Auxin Transport. doi:10.1371/journal/pbio.1000516**

